# The co-existence of NS5A and NS5B resistance-associated substitutions is associated with virologic failure in Hepatitis C Virus genotype 1 patients treated with sofosbuvir and ledipasvir

**DOI:** 10.1371/journal.pone.0198642

**Published:** 2018-06-01

**Authors:** Seiichi Mawatari, Kohei Oda, Kazuaki Tabu, Sho Ijuin, Kotaro Kumagai, Kunio Fujisaki, Masafumi Hashiguchi, Yukiko Inada, Hirofumi Uto, Yasunari Hiramine, Takeshi Kure, Takeshi Hori, Oki Taniyama, Ai Kasai, Tsutomu Tamai, Akihiro Moriuchi, Akio Ido

**Affiliations:** 1 Digestive and Lifestyle Diseases, Department of Human and Environmental Sciences,Kagoshima University Graduate School of Medical and Dental Sciences, Sakuragaoka, Kagoshima, Japan; 2 Department of HGF Tissue Repair and Regenerative Medicine, Kagoshima University Graduate School of Medical and Dental Sciences, Sakuragaoka, Kagoshima, Japan; 3 Department of Gastroenterology and Hepatology, Kagoshima City Hospital, Uearata-cho Kagoshima, Japan; 4 Department of Hepatology, Kirishima Medical Center, Kirishima, Hayato-cho, Kirishima, Kagoshima, Japan; 5 Center for Digestive and Liver Diseases, Miyazaki Medical Center Hospital, Takamatsu-cho, Miyazaki, Japan; 6 Department of Internal Medicine, Kagoshima Kouseiren Hospital, Tenpozan-cho, Kagoshima, Japan; Nihon University School of Medicine, JAPAN

## Abstract

**Objective:**

The present study aimed to reveal the factors associated with virologic failure in sofosbuvir and ledipasvir (SOF/LDV)-treated patients, and identify baseline NS5A or NS5B resistance-associated substitutions (RASs).

**Methods:**

Four hundred ninety-three patients with Hepatitis C Virus (HCV) genotype 1b infection were treated with SOF/LDV; 31 had a history of interferon (IFN)-free treatment with daclatasvir and asunaprevir. The effect of baseline RASs on the response to SOF/LDV therapy was analyzed.

**Results:**

Overall, a sustained virologic response at 12 weeks (SVR12) was achieved in 476 patients (96.6%). The SVR12 rates in the patients with IFN-free treatment-naïve and retreatment were 97.6% and 80.6%, respectively. HCV elimination was not achieved in 17 patients, 11 (including 5 with IFN-free retreatment) of whom had virologic failure. Eight patients had coexisting NS5A RASs of Q24, L28 and/or R30, L31, or Y93 and one patient had coexisting NS5A RASs of P32L and A92K. Interestingly, 10 and 8 patients had NS5B A218S and C316N RAS respectively. According to a multivariate analysis, coexisting NS5A RASs, NS5A P32 RAS, NS5B A218 and/or C316 RASs, and γ-glutamyltranspeptidase were associated with virologic failure. In the naïve patients, all patients without NS5B A218 and/or C316 RAS achieved an SVR12. Notably, the SVR12 rates of patients with coexisting NS5A and NS5B RASs were significantly lower (83.3%).

**Conclusions:**

Although SOF/LDV therapy resulted in a high SVR12 rate, coexisting NS5A and NS5B RASs were associated with virologic failure. These results might indicate that the coexisting baseline RASs influence the therapeutic effects of SOF/LDV.

## Introduction

Direct acting antiviral (DAA) therapy is associated with a high sustained virologic response (SVR) rate in patients with chronic hepatitis type C in comparison to conventional interferon (IFN) therapy. In July 2014, daclatasvir (DCV) (a NS5A inhibitor) and asunaprevir (ASV) (an NS3-4A protease inhibitor) were authorized for the treatment of chronic hepatitis or compensated cirrhosis in patients with hepatitis C virus (HCV) genotype 1 in Japan. It was reported that baseline resistance-associated substitutions (RASs) of NS3 D168, NS5A L31 and Y93 predicted the effects of DAA treatment in patients undergoing DCV/ASV combination therapy[1]. We previously reported that in patients without NS5A L31 or Y93 RASs and a history of protease inhibitor therapy, the virologic effect of DCV+ASV therapy was associated with the presence of NS5A Q24, L28, and/or R30 RASs and concomitant F37 and Q54 RASs before treatment[2].

In July, 2015, sofosbuvir (SOF) (an NS5B inhibitor) and ledipasvir (LDV) (an NS5A inhibitor) combination therapy were authorized for the treatment of chronic hepatitis or compensated cirrhosis in patients with HCV genotype 1 in Japan. An open-label phase 3 clinical trial in Japan showed that an SVR was achieved by 100% of patients [3]. The presence of baseline NS5A RASs did not affect the treatment outcome [4]. However, in the real world, some patients experience virologic failure. We therefore aimed to reveal the factors associated with virologic failure in patients treated with SOF/LDV, and to identify the baseline NS5A and NS5B RASs.

## Materials and methods

### The study population

A total of 493 patients with HCV genotype 1 infection who were treated with SOF (90 mg/day) and LDV (400 mg/day; Harboni; Gilead Sciences, Tokyo, Japan) for 12 weeks were followed for an additional 12 weeks after treatment. The study protocol conformed to the ethical guidelines of the Declaration of Helsinki and was approved by Kagoshima University Hospital Clinical Research Ethics Committee (approval number: 170199). Written informed consent was obtained from each patient. The HCV RNA concentration was measured by a TaqMan PCR, which has a lower quantitation limit of 1.2 log IU/mL. The FIB4 index, a surrogate marker of liver fibrosis, was calculated based on the methods of previous studies [5].

### The detection of HCV RASs

As described in detail previously [2], we investigated the viral genome sequence by direct sequencing. Total RNA was extracted from 140 μL of each serum sample using a QIAamp Viral RNA Mini Kit (QIAGEN, Tokyo, Japan). The extracted RNA was reverse-transcribed using a PrimeScript RT Reagent Kit attached Random 6 mers and Oligo dT Primer (TaKaRa Bio, Kusatsu, Japan). The target HCV genome was amplified by a nested PCR using PrimeSTAR GXL DNA Polymerase or LA Taq with GC Buffer (TaKaRa Bio). The primers used to amplify the NS5A and NS5B regions have been reported previously[6–9] (S1 Table). Reverse transcription was performed at 37°C for 15 minutes and terminated at 85°C for 5 seconds, followed by the first-round PCR over 30 cycles, with each cycle consisting of denaturation at 98°C for 10 seconds, annealing at 60°C for 15 seconds and extension at 68°C for 60 seconds. The second-round PCR was performed under the same condition. After purification with a GenElute PCR Clean-Up Kit (Sigma-Aldrich, Saint Louis, MO, USA), the nucleotide sequences of the second amplicons were determined using a BigDye Terminator v3.1 Cycle Sequencing Kit (Thermo Fisher Scientific, Waltham, MA, USA) and Sanger sequencing. The sequences of the NS5A and NS5B RAS positions in the HCV gene were determined using HCV-Con1 (accession no. AJ238799) as a reference, as previously described and the package insert of Harboni^®^ [10–18]; specifically, 10 NS5A baseline RASs (Q24/K/H/R, L28M/V/I/T, R30Q/L/H/G/E, L31M/V/I/F, P32L, F37L/I/Y/V/W, Q54H/Y/E/L/N/S, P58S/H/Q/L/A, A92T/E/V/K/S and Y93H/N/S/C/F), and 7 NS5B baseline RASs (L159F, A207T/I, A218S, S282T, C316N/H/Y, L320F, V321A/I) were investigated. If >20% of minor variants were detected in the virus population, it was regarded as RAS-positive.

### Statistical analysis

Statistical analyses were performed using the Statistical Package for Social Sciences software program (version 18, SPSS, Inc., Chicago, IL, USA). Categorical data were compared using the chi-squared test and Fisher's exact test, as appropriate. Continuous variables were analyzed using the Mann-Whitney U test. P values of <0.05 were considered to indicate statistical significance. Factors associated with virologic failure were determined using a multivariable logistic regression analysis with forward selection using p<0.15 as a cutoff for inclusion in the model. SVR was estimated in an intention-to-treat analysis.

## Results

### The baseline characteristics of the patients

The demographic baseline characteristics of the 493 patients are shown in Table 1. The age of the patients ranged from 26 to 90 years (mean, 68 years). There were 295 female patients (60%) and 105 (21%) patients with cirrhosis. Thirty-one patients had previously been treated with DCV and ASV. Sixty-three patients (13%) had previously been treated for hepatocellular carcinoma (HCC).

**Table 1:** The baseline characteristics of the patients who were treated with sofosbuvir/ledipasvir (n = 493).

Characteristics	IFN-free naïve (n = 462)	IFN-free retreatment (n = 31)	Total (n = 493)
Age, years (range)	68.1±10.3 (26–90)	69.5±8.7 (54–86)	68.2±10.2 (26–90)
Female, n (%)	272 (59)	23 (74)	295 (60)
Liver cirrhosis, n (%)	90 (20)	15 (48)	105 (21)
Previously treated hepatocellular carcinoma, n (%)	58 (13)	5 (16)	63 (13)
Previous treatment Naïve / IFN / IFN+RBV / PEG-IFN / PR / PR+PI / DCV+ASV / unknown, n	272 / 24 / 12 / 11 / 89 / 38 / 0 / 16	0 / 0 / 0 / 0 /0 / 0 / 31 / 0	272 / 24 / 12 / 11 / 89 / 38 / 31 / 16
Laboratory data			
HCV-RNA, log IU/mL	6.0±0.9	6.0±0.6	6.0±0.8
White Blood Cell, /μL	4741±1477	4001±1231	4695±1473
Hemoglobin, g/dL	13.6±1.5	13.0±1.4	13.6±1.5
Platelet, ×10^4^/μL	15.3±6.0	12.5±6.6	15.1±6.1
AST, IU/L	50±34	52±23	50±33
ALT, IU/L	47±39	46±25	47±38
GGT, IU/L	44±54	42±34	44±53
Total bilirubin, mg/dL	0.9±0.4	0.9±0.4	0.9±0.4
Albumin, g/dL	4.1±0.4 (n = 450)	4.0±0.4 (n = 29)	4.1±0.4 (n = 479)
α-Fetoprotein, ng/mL	11.7±33.2 (n = 450)	21.3±48.0	12.3±34.3 (n = 481)
Fib-4 index	4.23±3.33	5.60±3.48	4.32±3.35

IFN, interferon; RBV, ribavirin; PEG-IFN, pegylated-IFN; PR, PEG-IFN and RBV; PI, protease inhibitor; DCV+ASV, daclatasvir+asunaprevir; IL28B SNP, Interleukin 28B single nucleotide polymorphism; AST, aspartate transaminase; ALT, alanine aminotransferase; GGT, γ-glutamyltransferase. Data are shown as the mean ± SD unless otherwise indicated.

### The prevalence of baseline RASs in the study population

We investigated the prevalence of ten NS5A and seven NS5B baseline RASs in the study population (S2 Table). With the exception of A207, there was no significant difference in the NS5B RASs of the IFN-free treatment-naïve and IFN-free retreatment patients.

We investigated the co-existence of NS5A and NS5B RASs. Sixty of 87 patients (69.0%) had co-existing RASs of Q24, L28 or R30. Furthermore, 208 of 210 patients with NS5B C316 RAS (99.0%) had concomitant NS5B A218 and C316 RASs. In addition, 208 of 263 patients with NS5B A218 RAS (79.1%) had concomitant NS5B A218 and C316 RASs (It was not possible to detect A218 or C316 RASs in 11 patients).

### The virologic response rate

**Fig 1:**
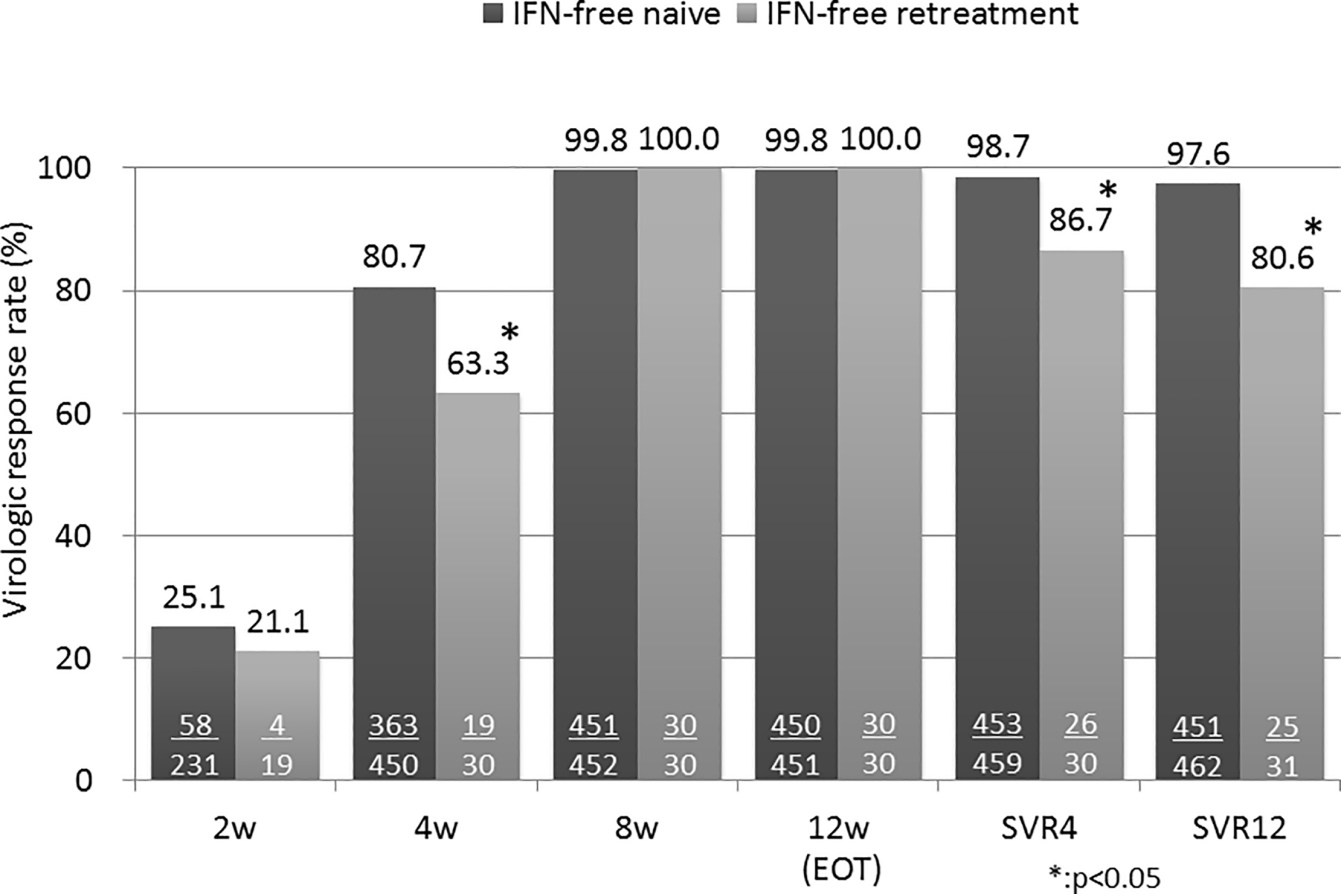
The virologic response rates (intention-to-treat analysis). The sustained virologic response (SVR) 12 rates of the IFN-free treatment-naïve patients and IFN-free retreatment patients were 97.6% and 80.6% respectively.

Among the 462 IFN-free treatment-naïve patients who received SOF/LDF, 452 patients (97.8%) completed treatment. Overall, 95.0% of the patients (363/450) had undetectable HCV RNA levels at the fourth week of treatment and 99.8% (450/451) had undetectable HCV RNA levels at the end of treatment. An SVR at 12 weeks (SVR12) was achieved in 451 patients (97.6%) in the intention-to-treat analysis (Fig 1). On the other hand, among the 31 patients who received SOF+LDF therapy in IFN-free retreatment, 30 patients (96.8%) completed treatment. Overall, 63.3% of patients (19/30) had undetectable HCV RNA levels at week 4 of treatment and 100% (30/30) had undetectable HCV RNA levels at the end of treatment. An SVR12 was achieved in 25 patients (80.6%) in the intention-to-treat analysis (Fig 1).

### The RASs in patients with virologic failure

Eleven (2.2%) patients (IFN-free treatment-naïve, n = 6; IFN-free retreatment, n = 5) were considered to have virologic failure (Table 2). All patients had relapsed disease. We examined the baseline RASs of these 11 patients. Eight of 11 patients (72.7%) had NS5A Y93 RASs, 5 of 11 patients (45.5%) had NS5A Q24, L28, and/or R30 RASs, and 4 of 11 patients (36.4%) had NS5A L31 RASs. Three patients had coexisting NS5A L31 and Y93 RASs at the baseline. In brief, 8 of 11 patients (72.2%) had coexisting NS5A Q24, L28 and/or R30 RASs, L31 RAS or Y93 RAS at the baseline. One patient who underwent IFN-free retreatment and who did not have NS5A L31 or Y93 RASs, had baseline NS5A P32L and A92K RASs.

**Table 2:** The baseline resistance-associated substitutions (RASs) and virologic failure in patients treated with sofosbuvir and ledipasvir.

Case	Age Gender	Etio- logy	Previous treatment (outcome)	RAS (upper row: baseline, lower: after virologic failure)																
				NS5A										NS5B						
				Q 24	L 28	R 30	L 31	P 32	F 37	Q 54	P 58	A 92	Y 93	L 159	A 207	A 218	S 282	C 316	L 320	V 321
1	62 F	CH	Naïve	K	M	Q	.	.	.	H/Q	.	.	Y/H	.	T	S	.	N	.	.
				K	M	Q	.	.	.	.	.	.	H	.	T	S	.	N	.	.
2	76 F	CH	PR (relapse)	K	M	Q	.	.	.	.	.	.	H	.	T	S	.	N	.	.
				K	M	Q	.	.	.	.	.	.	H	.	T	S	.	N	.	.
3	70 M	CH	Naïve	.	.	.	.	.	.	.	.	.	.	.	.	S	.	N	.	.
				.	.	.	V	.	.	.	.	.	H	.	.	S	.	N	.	.
4	56 F	LC	Naïve	.	.	.	.	.	.	Q/H	.	.	H	ND	.	S	.	N	.	.
				.	.	.	M	.	.	H	.	.	H	.	.	S	.	N	.	.
5	74 M	CH	Naïve	Q/R	.	Q	.	.	.	.	.	.	H	.	T	S	.	N	.	.
				.	.	Q	.	.	.	.	.	.	H	.	T	S	.	N	.	.
6	76 M	CH	PR (null)	.	.	.	M/L	.	L	.	.	.	H	ND	.	S	.	.	.	.
				.	.	.	M	.	L	.	.	.	H	.	.	S	.	.	.	.
7	57 F	CH	SMV +PR(null) DCV/ASV (null)	.	.	.	V	.	L	H	.	.	H	.	.	S	.	.	.	.
				.	.	.	V	.	L	H	.	.	H	.	.	S	.	.	.	.
8	73 F	CH	DCV/ASV (relapse)	.	.	.	.	L	L	H	.	K	.	ND	.	S	.	N	.	.
				.	.	.	.	L	L	.	.	K	.	.	.	S	.	N	.	.
9	80 F	LC	DCV/ASV (VBT)	K	M	Q	.	.	.	.	.	.	H	.	T	S	.	N	.	.
				K	M	Q	.	.	.	.	.	.	H	.	T	S	.	N	.	.
10	64 F	LC	DCV/ASV (null)	.	.	.	V	.	.	.	.	.	H	.	T	S	.	N	.	.
				.	.	.	V	.	.	.	.	.	H	.	T	S	.	N	.	.
11	86 M	CH	DCV/ASV (relapse)	.	M	.	F	.	L	H	L	.	.	.	.	.	.	.	.	.
				.	M	.	F	.	L	H	L	.	.	.	.	.	.	.	.	.

Dots mean non-RAS amino acid.

CH, chronic hepatitis; IL28B SNP, Interleukin 28B single nucleotide polymorphism; PR, pegylated-interferon and ribavirin; LC, liver cirrhosis; SNP, single nucleotide polymorphism; ND, no data; SMV, simeprevir; DCV, daclatasvir; ASV, asunaprevir; VBT, viral breakthrough.

In the analysis of post-treatment RASs, the patients who had coexisting Q24, L28, and/or R30, L31 or Y93 RASs at the baseline showed no change in their NS5A RAS profile. However, two patients acquired NS5A L31 and Y93 RASs. The patient who had baseline NS5A P32L and A92K RASs did not acquire NS5A L31 or Y93 RASs.

We analyzed 7 NS5B RASs. Interestingly, 10 of 11 patients had an NS5B A218 RAS, 8 of 11 patients had an NS5B C316 RAS, and 5 of 11 patients had an NS5B A207 RAS at the baseline. No patients had NS5B L159, S282, L320 or V321 RASs at the baseline. In particular, the patients who had NS5B C316 RAS also had coexisting A218 RAS. In addition, no patients acquired these RASs.

### Factors associated with virologic failure

We analyzed the factors associated with virologic failure among the patients who completed treatment. Among the blood biochemistry data, significant differences were observed in the platelet count and γ- glutamyltransferase (GGT) levels between the SVR12 and no-SVR12 groups; however, there were no differences between the IFN-free treatment-naïve and IFN-free retreatment groups (S3 Table).

Table 3 showed the SVR rates according to the various categories. A univariate analysis in all cases showed that HCV RNA ≥6.5 log IU/mL (p = 0.016), platelet count <10×10^4^/μL (p = 0.041), and GGT ≥37IU/L (p = 0.022) were associated with an SVR12. Among the RASs of NS5A, RASs of NS5A: Q24, L28, and/or R30 (p = 0.014), L31 (p = 0.006), P32 (p<0.001), and Y93 (p = 0.001), were associated with SVR12. In particular, coexisting NS5A RASs (Q24, L28, and/or R30, L31, or Y93 RAS) were associated with a lower SVR12 rate in comparison to no RASs or a single RAS. Interestingly, among the RASs of NS5B, RASs of A218 (p = 0.013) and C316 (p = 0.042) were associated with an SVR12. Similarly, NS5B A218 and/or C316 were significantly associated with an SVR12 (p = 0.015).

**Table 3:** The SVR12 rates of patients treated with ledipasvir and sofosbuvir.

Category	Cut off	IFN-free naïve (n = 452)			IFN-free retreatment (n = 30)			Overall (n = 482)		
		N	SVR (%)	*P* value	N	SVR (%)	*P* value	N	SVR (%)	*P* value
Age	<75	321	98.8	0.813	20	85.0	0.729	341	97.9	0.600
	≥75	131	98.5		10	80.0		141	97.2	
Gender	Male	187	98.4	0.666	7	85.7	0.847	194	97.9	0.790
	Female	265	98.9		23	82.6		288	95.6	
Etiology	CH	365	98.6	0.872	16	81.3	0.743	381	97.9	0.602
	LC	87	98.9		14	85.7		101	97.0	
Previously treated HCC	No	397	98.5	0.359	25	80.0	0.273	422	97.4	0.206
	Yes	55	100		5	100		60	100	
HCV-RNA	<6.5 logIU/mL	314	99.0	0.297	21	95.2	0.008	335	98.8	0.016
	≥6.5 logIU/mL	138	97.8		9	55.6		147	95.2	
Platelet	< 10.0 ×10^4^/μL	86	97.7	0.369	14	78.6	0.513	100	95.0	0.041
	≥10.0 ×10^4^/μL	366	98.9		16	87.5		382	98.4	
GGT	<37	272	99.6	0.028	20	90.0	0.166	426	98.4	0.022
	≥37	180	97.2		10	70.0		56	92.9	
Fib4-index	<6.55	383	99.0	0.215	20	85.0	0.729	403	98.3	0.070
	≥6.55	69	97.1		10	80.0		79	94.9	
NS5A:Q24, L28, and/or R30 RAS	No	375	99.2	0.023	18	83.3	1.000	393	98.5	0.014
	Yes	72	95.8		12	83.3		84	94.0	
NS5A:L31 RAS	No	411	98.8	0.435	14	85.7	0.743	425	98.4	0.006
	Yes	36	97.2		16	81.3		52	92.3	
NS5A:P32 RAS	No	446	98.7	0.907	28	85.7	0.190	474	97.9	<0.001
	Yes	1	100		2	50.0		3	66.7	
NS5A:F37 RAS	No	212	97.6	0.076	15	86.7	0.624	227	96.9	0.281
	Yes	235	99.6		15	80.0		250	98.4	
NS5A:Q54 RAS	No	242	98.3	0.535	12	83.3	1.000	254	97.6	0.931
	Yes	205	99.0		18	83.3		223	97.8	
NS5A:P58 RAS	No	415	98.6	0.493	27	85.2	0.414	442	97.7	0.821
	Yes	32	100		3	66.7		35	97.1	
NS5A:A92 RAS	No	413	98.5	0.479	26	84.6	0.631	439	97.7	0.889
	Yes	34	100		4	75.0		38	97.4	
NS5A:Y93 RAS	No	326	99.7	0.002	13	84.6	0.869	339	99.1	0.001
	Yes	122	95.9		17	82.4		139	94.2	
The number of NS5A RASs^†^	0	251	99.6	<0.001	5	80.0	0.399	256	99.2	<0.001
	1	161	99.4		7	100		168	99.4	
	≥2	34	88.2		18	77.8		52	84.6	
NS5B:L159 RAS	No	413	99.0	0.843	25	80.8	0.664	438	98.2	0.760
	Yes	4	100		1	100		5	100	
NS5B:A207 RAS	No	328	99.1	0.184	16	81.3	0.811	344	98.3	0.175
	Yes	117	97.4		13	84.6		130	96.2	
NS5B:A218 RAS	No	208	100	0.021	9	88.9	0.558	217	99.5	0.013
	Yes	237	97.5		20	80.0		257	96.1	
NS5B:S282 RAS	No	443	98.6		29	82.8		472	97.7	
	Yes	0	0		0	0		0	0	
NS5B:C316 RAS	No	257	99.6	0.039	13	84.6	0.811	270	98.9	0.042
	Yes	186	97.3		16	81.3		202	96.0	
NS5B:L320 RAS	No	443	98.6		29	82.8		472	97.7	
	Yes	0	0		0	0		0	0	
NS5B:V321 RAS	No	438	98.6	0.792	29	82.8		467	97.6	0.728
	Yes	5	100		0	0		5	100	
NS5B:A218 and/or C316 RASs	No	201	100	0.023	9	88.9	0.558	210	99.5	0.015
	Yes	235	97.4		20	80.0		255	96.1	

CH, chronic hepatitis; LC, liver cirrhosis; IL28B SNP, Interleukin 28B single nucleotide polymorphism; GGT, γ-glutamyltransferase; HCC, hepatocellular carcinoma; RAS, resistance-associated substitution.

^t†^ The number of NS5A RASs indicates the sum of Q24, L28, and/or R30 RAS, L31 RAS,and Y93 RAS.

Among the demographic parameters in the IFN-free treatment-naïve, RASs of NS5A Q24, L28, and/or R30 (p = 0.023), Y93 (p = 0.002), NS5B: A218 (p = 0.021) and C316 (p = 0.039) were associated with an SVR12. Similarly, coexisting NS5A RASs (Q24, L28, and/or R30, L31, or Y93 RAS) and NS5B RAS (A218 and/or C316) were associated with a lower SVR12 rate in comparison to no RASs or a single RAS. On the other hand, among the demographic parameters in the IFN-free retreatment group, IL28B SNP (p = 0.016), and HCV RNA ≥6.5 log IU/mL (p = 0.008), were associated with an SVR12; however, RASs of NS5A, and NS5B, were not associated with an SVR12.

Many patients with virologic failure had coexisting NS5A or NS5B RASs, we conducted a multivariable logistic regression analysis considering the coexistence of NS5A or NS5B RASs. More than 2 NS5A RASs (odds ratio [OR], 42.297; 95% confidence interval [CI], 6.250–286.241), NS5A P32 RAS (OR, 36.725; CI, 1.603–841.157), NS5B A218 and/or C316 RAS (OR, 19.377; CI, 1.597–235.071), and GGT ≥37IU/L (OR, 11.573; CI, 2.151–62.270) were also associated with virologic failure (Table 4).

**Table 4:** The multivariate logistic regression analysis of factors associated with virologic failure in patients treated with sofosbuvir and ledipasvir.

Category	Cut off	Univariate			Multivariate		
		OR	95% CI	*P* value	OR	95% CI	*P* value
Age	≥75 years	1.393	0.401–4.836	0.600			
Gender	Female	1.183	0.342–4.098	0.790			
HCV RNA	≥6.5 logIU/mL	4.138	1.192–14.359	0.016			
Platelet	<10×10^4^/μL	3.298	0.986–11.038	0.041			
GGT	≥37 IU/L	4.234	1.109–16.168	0.022	11.573	2.151–62.270	0.004
Fib-4 index	≥6.55	3.017	0.862–10.563	0.070			
The number of NS5A RAS^†^	≥2RASs	32.211	6.789–152.831	<0.001	42.297	6.250–286.241	<0.001
NS5A: P32 RAS	Yes	23.200	1.941–277.273	<0.001	36.725	1.603–841.157	0.024
NS5B:A207 RAS	Yes	2.253	0.676–7.514	0.175	0.306	0.057–1.643	0.167
NS5B:A218 and/or C316 RAS	Yes	8.531	1.083–67.192	0.013	19.377	1.597–235.071	0.020
IFN free therapy	retreatment	14.867	4.245–52.062	<0.001	3.017	0.590–15.425	0.185

OR, odds ratio; CI, confidence interval; IL28B SNP, Interleukin 28B single nucleotide polymorphism; GGT, γ-glutamyltransferase; RAS resistance-associated substitution; IFN, interferon

^†^The number of NS5A RASs indicates the sum of Q24, L28, and/or R30 RAS, L31 RAS, and Y93 RAS.

### SVR12 rate in patients with coexisting NS5A and NS5B RASs

**Fig 2:**
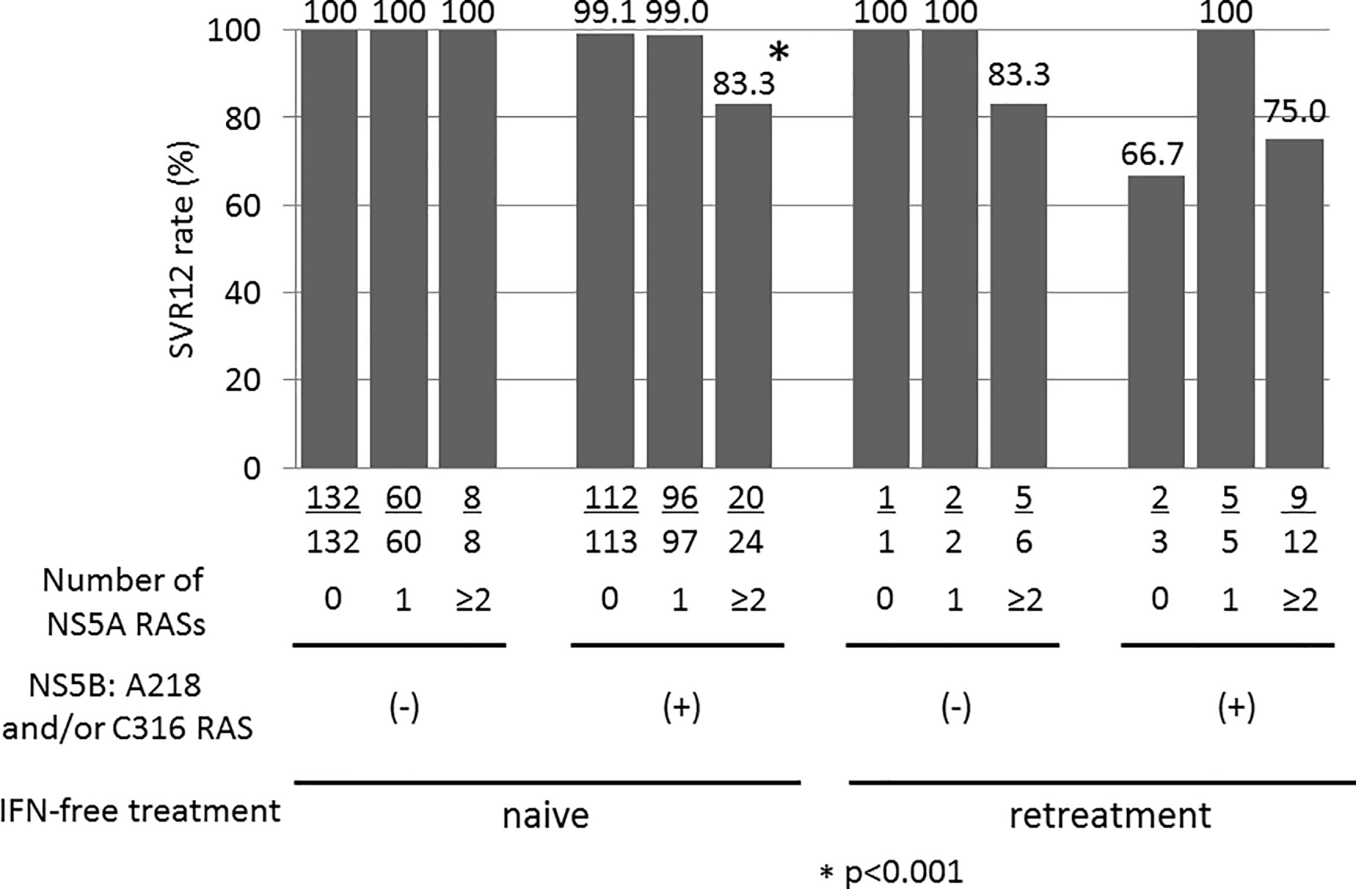
The sustained virologic response (SVR) 12 rate in the patients with co-existing NS5A and NS5B RASs. The interferon (IFN) free-naïve patients with coexisting resistance-associated substitutions (RASs) of Q24, L28, and/or R30, L31, or Y93 and NS5B A218 and/or C316 had a significantly lower sustained virologic response (SVR) 12 rate in comparison to the patients without these RASs.

We analyzed the SVR12 rate in patients with coexisting NS5A and NS5B RASs. Among the IFN-free treatment-naïve patients, all of the patients without NS5B A218 and/or C316 RASs achieved an SVR12 (Fig 2). In particular, the SVR12 rate of the patients who had more than two NS5A RASs (Q24, L28 and/or R30 RAS, L31 RAS, or Y93 RAS) and NS5B A218 and/or C316 RASs was 83.3%. On the other hand, in the IFN-free retreatment patients, all patients with virologic failure had coexisting NS5A RASs (Q24, L28 and/or R30 RAS, L31 RAS, or Y93 RAS) with the exception of one case that had NS5A P32L and A92K RASs; however these RASs did not have a significant difference on the SVR12 rate.

### Discontinuation of therapy

Eleven patients (2.2%) discontinued SOF/LDV therapy (Table 5). All patients were >65 years of age. Three patients discontinued therapy due to fatigue, and 2 patients each discontinued therapy due to renal injury or skin itching. Five patients discontinued therapy due to other reasons (hyperbilirubinemia, diarrhea, brain hemorrhage, and unknown). The mean time to discontinuation was 4.5 weeks. All patients receiving SOF/LDV for more than 8 weeks achieved an SVR12.

**Table 5:** The reasons for the discontinuation of sofosbuvir/lediprevir.

case	Age gender	Etiology	Previous treatment	Previously treated HCC	Discontinued weeks	Reason of discontinued therapy	outcome
1	77M	CH	Naïve	No	10	Unknown	SVR24
2	66M	CH	SMV+PR	No	9	Skin Itching	SVR24
3	90F	CH	Naïve	No	8	Skin Itching	SVR24
4	68M	LC	PR	No	8	Depression, fatigue	SVR24
5	78F	LC	Naïve	Yes	5	Brain hemorrhage	Relapse
6	79F	CH	Naïve	Yes	4	Renal injury	SVR24
7	74M	LC	DCV/ASV	No	2	Renal injury	Non-SVR
8	76F	CH	Naïve	No	1	Fatigue	Non-SVR
9	74F	LC	IFN+RBV	No	1	Hyperbilirubinemia	Non-SVR
10	72F	CH	IFN	Yes	1	Diarrhea	Non-SVR
11	77F	CH	Naïve	No	1	Fatigue	Non-SVR

HCC, hepatocellular carcinoma; CH, chronic hepatitis; SVR, sustained virologic response; SMV, simeprevir; PR, pegylated interferon and ribavirin; LC, liver cirrhosis; DCV, daclatasvir; ASV, asunaprevir; IFN, interferon; RBV, ribavirin.

## Discussion

We revealed, for the first time, that most SOF/LDV-treated patients with virologic failure had RASs of NS5B A218 and/or C316, and these RAS was significantly associated with virologic failure. Donaldson et al. reported that NS5B C316 was associated with virologic failure [17]; however while many studies have reported the analysis of large study populations [19–25], no reports have indicated that NS5B A218 RAS is associated with virologic failure in SOF/LDV-treated patients. The reason why we paid attention to NS5B A218 and/or C316 RAS was that 8 of the 11 patients with virologic failure had coexisting NS5B A218 and C316 RAS (Table 2). In addition, NS5B A218 and C316 showed a high rate of coexistence at baseline. Iio et al. showed a similar report about the coexistence of NS5B A218 and C316 in patients with virologic failure [19]. Donaldson et al. reported that NS5B C316N may interfere with the ability of SOF to enter the active site by blocking the space [17]; thus, these RASs might have inhibited the activity of SOF cooperatively.

The prevalence of NS5B A218 in the IFN-free treatment-naïve and IFN-free retreatment patients in this cohort was 53.6% (244/455) and 70.0% (21/30) respectively (S2 Table). Further, the prevalence of NS5B C316 in the IFN-free treatment-naïve and IFN-free retreatment patients in this cohort was 42.7% (193/452) and 56.7% (17/30) respectively (S2 Table). Ito et al. reported that the prevalence of NS5B C316N in 96 Japanese patients was 46.9% [9]. However, no studies have reported the prevalence of NS5B A218 RAS. Especially, 99.0% of patients who had NS5B C316 RAS had coexisting A218 RAS. The prevalence needs to be confirmed in other populations.

SOF is a nucleotide inhibitor directed at the activity of NS5B, the protein of which is essential for HCV replication. SOF, which prevents HCV RNA synthesis by acting as a “chain terminator”, has broader genotype coverage and a higher barrier to viral resistance [13]. Some clinical researchers reported that RASs of NS5B L159, S282, C316, L320, or V321 were associated with virologic failure [14, 15, 17]. However, with the exception of S282, these RASs were associated with low-level resistance in the replicon systems [13, 14, 16, 26]. In this cohort, no cases had RASs of S282, L320 before treatment or at the time that treatment was deemed unsuccessful, and all of the cases with RASs of L159 and V321 achieved an SVR (Table 3). There have been no reports about the RAS of NS5B A218 or the coexistence of A218 and C316 *in vitro*. Thus, it is important to confirm the effects of SOF in cases involving the coexistence of NS5B A218 and/or C316 RASs using a replicon system or a human hepatocyte chimera mouse model as soon as possible.

SOF/LDV therapy was associated with good therapeutic outcomes, even in patients with NS5A Y93 RAS, with 100% of the patients in a phase III trial in Japan showing an SVR [3]. Some reports indicate that NS5A Y93 RAS is not related to the therapeutic effect [4, 19, 24]. The American Association for the Study of Liver Diseases/Infectious Diseases Society of America [27] and the European Association for the Study of the Liver guidelines [28] have not mentioned any need for the routine investigation of NS5A RAS. On the other hand, other reports suggest that it is related to the therapeutic effect [20, 21]. In the present study, the SVR12 rate was 97.6%; thus the routine investigation of NS5A RAS might not be necessary. However, most cases of virologic failure involved coexisting NS5A RAS, and this was related to the therapeutic effect (Table 4). It is noteworthy that the SVR rate of the IFN free-naïve patients with a single substitution of Q24, L28, and/or R30, L31, or Y93 were significantly higher than those with more than two substitution (>99% vs 88.2%) (Table 3), and most cases of virologic failure involved the coexistence of these RASs (Table 2). On the other hand, an SVR was achieved in all cases without NS5B A218 and/or C316 RASs. In contrast, the SVR12 rates of the patients who had more than two NS5A RASs and NS5B A218 and/or C316 RAS was 83.3% (Fig 2). It was suggested that the coexistence of NS5A and NS5B RASs was highly associated with virologic failure; thus, investigating the baseline NS5A and NS5B RAS status, is useful for in efforts to increase the SVR rate to 100%. Therefore, the existence of baseline RAS should be considered at the choice of treatment regimen in order to eradicate HCV.

Overall, HCV elimination is not achieved in 10–15% of the patients who receive DCV/ASV [1, 2, 29]. This represented a major problem for HCV treatment because such patients had coexisting RASs in the NS3 and NS5A regions and these RASs had cross-resistance [30]. Upon DCV/ASV treatment failure in human hepatocyte chimeric mice, NS5A L31V and Y93H RAS were relatively resistant to ledipasvir and NS5B polymerase nucleotide inhibitor [31]. In previous studies, the SVR rates of IFN-free retreatment patients who received LDV/SOF (+ribavirin) were 71–86.7% [20, 32–34]. However, it was unclear which RASs were associated with virologic failure in these patients. In the present study, most patients with a single RAS of NS5A were achieved an SVR; however the result did not reach statistical significance, because of the small number of cases. Furthermore, NS5A P32 RAS was observed in 3 patients (one patient was IFN-free treatment-naïve, two patients received IFN-free retreatment). IFN-free treatment-naïve patients with NS5A P32 RAS achieved an SVR, one of two IFN-free retreatment patients showed virologic failure and had coexisting NS5A A92K RAS. These RASs were very rare; however, they have been reported to emerge after failure to respond to DCV/ASV [2, 29, 35]. NS5A A92K RAS is reported to have over 1000-fold resistance to LDV in comparison to the wild-type; in addition, P32L RAS have 2.5–10.0-fold resistance [26]. The coexistence of NS5A P32L and A92K RASs might be associated with the therapeutic effect. Meanwhile, there were no patients with NS5A P32 deletion in this cohort. In Japan, Glecaprevir/Pibrentasvir therapy was approved in September 2017, following a study in which the SVR rate in patients with DAA retreatment was 93.9%; however, two patients with virologic failure had an NS5A P32 deletion [36]. In the future, it is necessary to clarify the factors associated with the therapeutic effects of IFN-free retreatment patients in a large study population.

In the present study, GGT elevation was associated with virologic failure. Abe et al. reported that GGT elevation was associated with IL28B polymorphism [37]. The therapeutic effect of SOF/LDV might be related to host factors in IFN-free retreatment especially.

In the present study, 11 patients (2.2%) discontinued SOF/LDV therapy. In previous studies, 0–5.3% of patients who received SOF/LDV discontinued therapy [19–25]. All patients were >65 years of age. Thus physicians should pay careful attention when SOF/LDV is administered to elderly patients.

The present study was associated with several limitations. First, only 11 (2.2%) of 482 patients had virologic failure; thus, the range of the 95% CI was wide, despite the fact that a statistically significant difference was observed. Second, it was not possible to detect all RASs in some cases. Third, since the number of cases was smaller than that of previous studies, a larger cohort should be analyzed in a future study.

In conclusion, SOF/LDV therapy showed a high SVR12 rate. However, the coexistence of NS5A and NS5B RASs were associated with virologic failure. These results might indicate that the coexistence of baseline RASs influence the therapeutic effects of SOF/LDV.

## Supporting information

S1 Table Amplification and sequencing primers used in this study. (DOCX)

S2 Table Prevalence of resistance-associated substitutions (RASs) in the study population. (DOCX)

S3 Table The baseline characteristics according to the achievement/non-achievement of an SVR in the patients completing SOF/LDV therapy. (DOCX)

S4 Table Analysis data set. All patients data sets were included in the following file.
(XLSX)
