# Supplementary material for: The co-existence of NS5A and NS5B resistance-associated substitutions is associated with virologic failure in Hepatitis C Virus genotype 1 patients treated with sofosbuvir and ledipasvir
Source: PLoS One. 2018 Jun 1;13(6):e0198642. doi: 10.1371/journal.pone.0198642 (PMC5983500; doi:10.1371/journal.pone.0198642)
Supplement: S1 Table — (DOCX) [file pone.0198642.s001.docx]

S1 Table: Amplification and sequencing primers used in this study

| Primer set | Primer name | Primer sequence | Strand | Position |
| --- | --- | --- | --- | --- |
| 1[6,7] | NS5A-F1 | 5'-TACTCCCTGCCATCCTCTCTCCTG-3' | sense | 5974-5997 |
|  | NS5A-F2 | 5'-CTCCTTGAGCACGTCCCGGT-3' | antisense | 7777-7796 |
|  | NS5A-F3 | 5'-TCTCCAGCCTTACCATCACYCA-3' | sense | 6172-6193 |
|  | NS5A-F4 | 5'-CGGTARTGRTCGTCCAGGAC-3' | antisense | 7761-7780 |
| 2[8] | 5A-1-N1 | 5'-ATGAACCGRCTGATAGCGTT-3' | sense | 6075-6094 |
|  | 5A-1b-N2R | 5'-CTAGCTGAAGAGCTGGCCAA-3' | antisense | 6921-6940 |
|  | 5A-1-N3 | 5'-TCCCCYACRCACTATGTGCC-3' | sense | 6117-6136 |
|  | 5A-1B-N4R | 5'-CGCTTRGCCGTYTCTGCTGT-3' | antisense | 6873-6892 |
| 3[9] | NS5B 1st sense | 5'-GGCTAYGGGGCAAAGGACGTC-3' | sense | 7902-7922 |
|  | NS5B 1st antisense | 5'-GCYGGRATTGGAGTGAGTTTRAG-3' | antisense | 9198-9220 |
|  | NS5B 2nd sense forward | 5'-CTCCGTGTGGAAGGACYTGC-3' | sense | 7958-7977 |
|  | NS5B 2nd antisense reverse | 5'-TACCTRGTCATRGCCTCCGTG-3' | antisense | 8615-8635 |
| 4 | NS5B 1bF1 | 5'-CAACTCTTTGCTGCGTCACC-3' | sense | 7679-7698 |
|  | NS5B 1bR1 | 5'-AAGCTTGGTCCTTACTGCCC-3' | antisense | 9181-9200 |
|  | NS5b 1bF2 | 5'-GCGTCCAACCAGAGAAAGGA-3' | sense | 8035-8054 |
|  | NS5b 1bR2 | 5'-TGTTGCCTAGCCAGGAGTTG-3' | antisense | 8803-8832 |

The position of primer set 1 and 2 refer to HCV-J (accession no. D 90208), and primer set 3 and 4 refer to HCV-Con 1 (accession no AJ 238799).
