# Supplementary material for: The co-existence of NS5A and NS5B resistance-associated substitutions is associated with virologic failure in Hepatitis C Virus genotype 1 patients treated with sofosbuvir and ledipasvir
Source: PLoS One. 2018 Jun 1;13(6):e0198642. doi: 10.1371/journal.pone.0198642 (PMC5983500; doi:10.1371/journal.pone.0198642)
Supplement: S2 Table — (DOCX) [file pone.0198642.s002.docx]

S2 Table: Prevalence of resistance-associated substitutions (RASs) in the study population

|  | RAS | IFN free naïve (n=462) | IFN free retreatment (n=31) | P value |
| --- | --- | --- | --- | --- |
| NS5A | Q24 | 53 (11.6) (n=457) | 8 (25.8) | 0.021 |
|  | L28 | 45 (9.8) (n=457) | 11 (35.5) | <0.001 |
|  | R30 | 66 (14.4) (n=457) | 9 (29.0) | 0.029 |
|  | L31 | 38 (8.3) (n=457) | 17 (54.8) | <0.001 |
|  | P32 | 1 (0.2) (n=456) | 2 (6.5) | <0.001 |
|  | F37 | 240 (52.5) (n=457) | 16 (51.6) | 0.922 |
|  | Q54 | 210 (46.0) (n=457) | 19 (61.3) | 0.098 |
|  | P58 | 32 (7.0) (n=457) | 3 (9.7) | 0.576 |
|  | A92 | 36 (7.9) (n=457) | 4 (12.9) | 0.324 |
|  | Y93 | 123 (26.9) (n=458) | 18 (58.1) | <0.001 |
| NS5B | L159 | 4 (0.9) (n=426) | 1 (3.7) (n=27) | 0.182 |
|  | A207 | 121 (26.6) (n=455) | 13 (43.3) (n=30) | 0.047 |
|  | A218 | 244 (53.6) (n=455) | 21 (70.0) (n=30) | 0.081 |
|  | S282 | 0 (0) (n=453) | 0 (0) (n=30) |  |
|  | C316 | 193 (42.7) (n=452) | 17 (56.7) (n=30) | 0.135 |
|  | L320 | 0 (0) (n=452) | 0 (0) (n=30) |  |
|  | V321 | 5 (1.1) (n=452) | 0 (0) (n=30) | 0.563 |

RAS resistance-associated substitution
