# Supplementary material for: The co-existence of NS5A and NS5B resistance-associated substitutions is associated with virologic failure in Hepatitis C Virus genotype 1 patients treated with sofosbuvir and ledipasvir
Source: PLoS One. 2018 Jun 1;13(6):e0198642. doi: 10.1371/journal.pone.0198642 (PMC5983500; doi:10.1371/journal.pone.0198642)
Supplement: S3 Table — (DOCX) [file pone.0198642.s003.docx]

S3 Table: The baseline characteristics according to the achievement/non-achievement of an SVR

| Characteristics | IFN free naïve (n=452) | | | IFN free retreatment (n=30) | | | Overall (n=482) | | |
| --- | --- | --- | --- | --- | --- | --- | --- | --- | --- |
|  | SVR | Non-SVR | *P* value | SVR | Non-SVR | *P* value | SVR | Non-SVR | *P* value |
|  | (n=446) | (n=6) |  | (n=25) | (n=5) |  | (n=471) | (n=11) |  |
| Age, years | 67.9±10.4 | 69.0±8.3 | 0.855 | 68.8±8.3 | 72.0±11.7 | 0.522 | 68.0±10.3 | 70.4±9.6 | 0.531 |
| HCV RNA, log IU/mL | 6.0±0.9 | 6.2±0.7 | 0.625 | 6.0±0.6 | 6.4±0.5 | 0.131 | 6.0±0.8 | 6.3±0.6 | 0.191 |
| White blood cell count, /μL | 4758±1478 | 4655±893 | 0.940 | 4005±1317 | 4042±967 | 0.867 | 4718±1479 | 4376±935 | 0.477 |
| Hemoglobin, g/dL | 13.6±1.5 | 13.9±1.0 | 0.733 | 13.3±1.4 | 12.9±1.0 | 0.358 | 13.6±1.5 | 13.4±1.1 | 0.588 |
| Platelet count, ×10^4^/μL | 15.4±6.0 | 12.4±5.7 | 0.337 | 13.0±.7.0 | 9.7±4.9 | 0.344 | 15.2±6.0 | 11.2±5.3 | 0.038 |
| AST, IU/L | 50±34 | 51±29 | 0.974 | 53±22 | 51±32 | 0.656 | 51±33 | 51±29 | 0.987 |
| ALT, IU/L | 48±39 | 54±36 | 0.705 | 47±24 | 50±34 | 0.911 | 48±38 | 52±34 | 0.651 |
| GGT, IU/L | 44±53 | 88±103 | 0.088 | 40±35 | 54±31 | 0.276 | 44±52 | 73±78 | 0.036 |
| Total bilirubin, mg/dL | 0.9±0.4 | 1.0±0.5 | 0.669 | 1.0±0.4 | 0.7±0.3 | 0.187 | 0.9±0.4 | 0.9±0.4 | 0.706 |
| Albumin, g/dL | 4.1±0.4 (n=434) | 4.0±0.5 | 0.899 | 4.0±0.4 (n=23) | 3.9±0.2 | 0.262 | 4.1±0.4 (n=457) | 4.0±0.4 | 0.339 |
| α-fetoprotein, ng/mL | 11.4±33.1 (n=434) | 21.6±42.0 | 0.698 | 22.4±53.0 | 18.8±19.4 | 0.119 | 12.0±34.4 (n=459) | 20.3±32.2 | 0.314 |
| Fib-4 index | 4.17±3.23 | 5.26±4.44 | 0.521 | 5.49±3.63 | 6.22±3.40 | 0.522 | 4.24±3.26 | 5.70±3.84 | 0.122 |

IFN, interferon; SVR, sustained virologic response; AST: aspartate transaminase; ALT, alanine aminotransferase; GGT, γ-glutamyltransferase
